# Getting the optimal technique for EUS-guided liver biopsy: are we there?

**DOI:** 10.1093/gastro/goaf063

**Published:** 2025-06-26

**Authors:** Gabrielle Sanford, Qiang Cai

**Affiliations:** Division of Gastroenterology and Hepatology, Louisiana State University Health Sience Ceter, Shreveport, LA, USA; Division of Gastroenterology and Hepatology, Louisiana State University Health Sience Ceter, Shreveport, LA, USA

In this issue, the animal study conducted by Ying Wu *et al.* on optimizing techniques and specimen quality in endoscopic ultrasound-guided liver biopsy (EUS-LB) is a meticulous and compelling contribution to the field [1]. Liver biopsy (LB) remains the gold standard for the diagnosis of liver disease. Traditionally, there are two non-surgery ways of performing a LB: percutaneous liver biopsy (PLB) and transjugular liver biopsy (TJLB). The former can be performed at the bedside, either through percussion of the right hepatic lobe or with the aid of pre-biopsy ultrasound guidance or real-time imaging to minimize complications and enhance results. On the other hand, TJLB is performed by interventional radiologists and is ideal for patients with certain conditions that are unsafe for PLB, such as ascites, coagulopathy, or obesity [2, 3]. In almost all cases, TJLB also samples the right hepatic lobe via the right hepatic vein, capitalizing on its anatomical access advantages.

EUS-LB is an emerging and promising technique for sampling liver parenchyma. Several studies have shown that EUS-LB has a good safety profile and increases patient comfort, as well as the ability to obtain large samples of tissue. Compared with PLB and TJLB, EUS-LB offers several distinct advantages: (i) it can biopsy both the right and left lobes, thus reducing or obviating sampling errors; (ii) it can biopsy both target lesions and non-target parenchymal; (iii) it enables simultaneous endoscopic evaluation, such as screening for esophageal varices or the evaluation of other upper gastrointestinal pathology; (iv) it facilitates elastography and direct portal pressure measurement in the same session; (v) it can reduce patient anxiety and improve patient satisfaction, as it is performed under sedation; (vi) it is associated with fewer complications, less post-procedure pain, and a shorter recovery time [2–9].

However, in the realm of liver histology, the method of biopsy does matter, as the adequacy of the sample is crucial. Adequacy is defined by several liver societies. For instance, the American Association for the Study of Liver Diseases defines an adequate LB sample as one that is 2–3 cm in length and contains at ≥11 complete portal tracts (CPTs) [10]. Over the past 15 years, EUS needles have continued to evolve from fine-needle aspiration (FNA) to spring-loaded biopsy to fine-needle biopsy (FNB). Current FNB needles with incorporated cutting surfaces allow reliable core tissue acquisition [10].

Despite the publication of several studies on EUS-LB in recent years, the optimal technique has not yet been definitively established [2–13]. Among the key factors influencing success are the selection of biopsy needles, preparation of the needle, and the application of optimal and safe needle biopsy techniques. The study conducted by Ying Wu *et al.* is timely and important in this regard. In their study, EUS-LBs were performed on four porcine subjects with the evaluation of four technical aspects: negative pressure suction, number of needle actuations, two puncture manipulations, and two types of puncture biopsy needles. The primary outcomes included total specimen length and the number of CPTs. The secondary outcomes included the longest specimen length, number of specimen pieces, specimen fragmentation, blood contamination, and bleeding. This study provides valuable evidence regarding optimal techniques for EUS-LB to acquire high-quality specimens. The conclusions of the study suggest that optimal techniques for specimen quality in EUS-LB include using 1-ml suction, limiting the procedure to no more than three actuations, and employing a modified “click-puncture” manipulation. Additionally, this study demonstrated that both FNA and FNB needles yield comparable specimen quality, with the FNA needle offering a slight advantage of preventing bleeding and foreign tissue embedding.

The author does acknowledge that there were several limitations to their study. The primary limitation was that the study was performed on animal models and the liver tissue from animals may differ from that of humans. Therefore, further studies on human subjects are needed to validate the conclusions of this study.

In conclusion, all three techniques (PLB, TJLB, EUS-LB) can be used obtain adequate liver specimens. EUS-LB would be the method of choice if there is available expertise. Based on current evidence regarding the optimal technique for EUS-LB, LB should be tailored to the individual patient and clinical indication.
